# The evolutionary costs of immunological maintenance and deployment

**DOI:** 10.1186/1471-2148-8-76

**Published:** 2008-03-03

**Authors:** Kurt A McKean, Christopher P Yourth, Brian P Lazzaro, Andrew G Clark

**Affiliations:** 1 Department of Biological Sciences, SUNY at Albany, Albany NY 12222, USA; 2 Department of Ecology and Evolutionary Biology, University of Toronto, Toronto ON M5S 3B2, Canada; 3 Department of Entomology, Cornell University, Ithaca NY 14853, USA; 4 Department of Molecular Biology and Genetics, Cornell University, Ithaca NY 14853, USA

## Abstract

**Background:**

The evolution of disease resistance and immune function may be limited if increased immunocompetence comes at the expense of other fitness-determining traits. Both the maintenance of an immune system and the deployment of an immune response can be costly, and the observed costs may be evaluated as either physiological or evolutionary in origin. Evolutionary costs of immunological maintenance are revealed as negative genetic correlations between immunocompetence and fitness in the absence of infection. Costs of deployment are most often studied as physiological costs associated with immune system induction, however, evolutionary costs of deployment may also be present if genotypes vary in the extent of the physiological cost experienced.

**Results:**

In this study we analyzed evolutionary and physiological costs of immunity in two environments representing food-limited and food-unlimited conditions. Patterns of genetic variation were estimated in females from 40 'hemiclone families' isolated from a population of *D. melanogaster*. Phenotypes evaluated included fecundity, weight measures at different time periods and resistance to *Providencia rettgeri*, a naturally occurring Gram-negative pathogen of *D. melanogaster*. In the food-limited environment we found a negative genetic correlation between fecundity in the absence of infection and resistance, indicative of an evolutionary cost of maintenance. No such correlation was observed in the food-unlimited environment, and the slopes of these correlations significantly differed, demonstrating a genotype-by-environment interaction for the cost of maintenance. Physiological costs of deployment were also observed, but costs were primarily due to wounding. Deployment costs were slightly exaggerated in the food-limited environment. Evolutionary costs of immunological deployment on fecundity were not observed, and there was only marginally significant genetic variation in the cost expressed by changes in dry weight.

**Conclusion:**

Our results suggest that the costs of immunity may be an important factor limiting the evolution of resistance in food-limited environments. However, the significant genotype-by-environment interaction for maintenance costs, combined with the observation that deployment costs were partially mitigated in the food-unlimited environment, emphasizes the importance of considering environmental variation when estimating patterns of genetic variance and covariance, and the dubious nature of predicting evolutionary responses to selection from quantitative genetic estimates carried out in a single environment.

## Background

Understanding factors affecting susceptibility to infectious disease is the goal of many branches of the biological sciences. For the evolutionary biologist, these factors include the forces of mutation, gene flow, recombination, drift and selection and how these processes have shaped, and are currently shaping, genetic variation contributing to susceptibility to parasites and pathogens. The continued maintenance of genetic variation for disease resistance present in most populations [1-6] poses an evolutionary problem; why, in the face of often strong pathogen mediated selection pressures, does disease susceptibility persist?

One hypothetical solution to this problem is that the rapid generation times of pathogens may provide them an evolutionary advantage over the host. Furthermore, for longstanding pathogen-host interactions, cycles of pathogen adaptation and host counter-adaptation may promote the rapid evolution of genes involved in defense and the maintenance of host polymorphisms through frequency dependent selection [7-9].

In addition to the assumed advantage of pathogens over their hosts, and potential pathogen-host coevolution, evolutionary and ecological immunologists have more recently begun to consider the consequences of the host life history on patterns of disease susceptibility [10-15]. The allocation of limited resources among fitness traits is a basic tenet of life history theory [16], and implicit in recognizing pathogen defense as an important fitness component is the understanding that if this defense is costly, the best genotype may not be the most resistant to disease, but the genotype making the best compromise with other fitness components such as growth, somatic maintenance and reproduction. Models of the pathogen-host interaction incorporating such costs suggest populations experience higher levels of disease susceptibility than would be seen if defenses were cost free [17,18].

Pathogen defense is a multifaceted trait, including behavioral, morphological and physiological components [14,19]. However, the outcome of the pathogen/host interaction often involves the immune system due to its specific role in combating potential pathogens that evade other defenses. Because of the central role of immune function in pathogen defense, resistance costs are often referred to as the costs of immunity.

The immune system of insects, while lacking the combinatorial specificity and antigenic memory characteristic of vertebrate immune function, is an effective mechanism of immunological defense providing protection against a wide variety of potential pathogens of bacterial, fungal, viral and multicellular parasitic origins. *Drosophila melanogaster *is a model system for much of our understanding of both constitutively expressed and inducible immune mechanisms including antimicrobial peptide production, phagocytosis of potential pathogens by insect hemocytes, the melanization reaction and encapsulation [14,20,21].

But what are the costs of immunity? First, we must distinguish between maintenance costs and deployment costs, and second, whether these costs are physiological or evolutionary [10]. Maintenance costs arise as a consequence of the investment of energy and resources into the infrastructure of an immune system and ongoing immunological surveillance and maintenance in the absence of infection. Such costs might arise if developing the organs necessary for immune surveillance, the maintenance of populations of cells necessary for immunity, or the production of constitutively expressed prophylactic measures including lysozyme and antimicrobial peptides, make energy and resources less available to other fitness promoting traits. Deployment costs arise from mounting an immune response. Such costs may be due to the utilization of energy or resources or as a consequence of immunopathology (collateral damage) associated with induction of an immune response. Indeed, models of the evolution of inducible defense require some cost, otherwise we would expect the constitutive expression of these defense mechanisms [22,23].

A second distinction must be made between physiological costs and evolutionary costs. Physiological costs are evaluated either by examining the phenotypic correlation between fitness traits or through experimental manipulation. For example, the experimental manipulation of male sexual activity results in a decline in their immune function, consistent with the hypothesis that the maintenance of an immune system carries some physiological cost [24]. Physiological costs of immunological deployment are evaluated as changes in fitness following experimental immunological challenge. The ease of inducing an immune response has prompted a number of experimental studies demonstrating physiological costs of immunological deployment [10,13].

Evolutionary costs imply an underlying genetic basis to the observed cost and are evolutionary in the sense that they may act as brakes retarding the response to selection for immune efficacy. Evolutionary costs of immunological maintenance are revealed as negative genetic correlations between immunocompetence and the expression of other fitness components in the absence of infection [11,14]. In *D. melanogaster*, for example, experimental evolution of resistance to the parasitoid wasps *Leptopilina boulardi *and *Asobara tabida *revealed a genetic trade-off with larval competitive ability in crowded larval conditions [2,4].

Evolutionary costs of deployment indicate that genotypes vary in cost of immune system induction. Such costs would be revealed either as a significant genotype-by-immune challenge interaction when comparing fitness traits in immune challenged versus unchallenged individuals or by demonstrating that costs of deployment are exaggerated as a consequence of selection on immune system function. There are no previous attempts to evaluate evolutionary costs of immunological deployment. In *D. melanogaster *immunological defense against the parasitoid *Asobara tabida *caused declines in desiccation and starvation resistance and the magnitude of this cost varied among iso-female lines [25], however, in this study it is impossible to distinguish immunological costs from costs associated with parasitism.

Environmental variation is known to affect the appearance and magnitude of both physiological and evolutionary costs of immunity. For example, reduced larval competitiveness in parasitoid-resistant *D. melanogaster *was only revealed in a highly competitive environment [2,4], while in bumblebees the costs of immune system activation were only apparent if workers were starved [26]. In general, environmental variation may affect condition and thus the amount of resource that can be allocated among various traits [27,28]. Furthermore, changes in genetic architecture are expected with environmental variation, affecting both the heritability of traits and the patterns of correlation between traits [29,30]. In particular, differences in the environmental lability of traits, affecting the extent of genotype-by-environment (GxE) interaction, are likely to influence the genetic correlation [31].

In this experiment we isolated 40 hemiclones from a natural population of *D. melanogaster *and used them to estimate the evolutionary costs of immunological maintenance and deployment under both food-limited and food-unlimited environmental conditions. We evaluated maintenance costs as the genetic correlation between fecundity in the absence of infection and resistance to an experimental infection with the bacterium *Providencia rettgeri*. We estimated deployment costs as the change in fecundity following infection with live bacteria or heat-killed bacteria in comparison with both sterile wounding and uninjected controls. Our results indicate the presence of maintenance costs, but that these costs can be wholly mitigated in an environment in which food resources are not limiting. We also detected deployment costs, but as with maintenance costs, the decline in fecundity due to immune challenge was condition dependent.

## Results

The final data set included counts of 6,425 vials totaling 892,682 emerging offspring, colony counts of *P. rettgeri *from 605 plates, and the dry weight of 5,248 females. Because of a labeling error, data for 2 of the hemiclone lines is missing from the fourth block of the experiment. Otherwise, sources of departure from the balanced design were random with respect to hemiclone line.

### Analysis of the cost of maintenance

Female fecundity in the absence of infection (vial 3 fecundity), resistance to *P. rettgeri*, dry weight at emergence, dry weight at day 9, and the change in dry weight from emergence to day 9 are included in the analysis of maintenance costs. Hemiclone lines varied for all of these traits in both environments (Table 1 and 2).

**Table 1:** Maintenance Cost: ANOVA summaries of Relevant Phenotypes

	Source		
	Hemiclone (HC)	Diet (D)	HC × D
Vial 3	3.49***	6452.91***	1.97***
Fecundity	(39,1484)	(1,1484)	(39,1484)
*P. rettgeri*	3.15***	0.39	1.28
Load	(39,518)	(1,518)	(39,518)
Day 9	11.54***	629.68***	2.52***
Weight	(39,1381)	(1,1381)	(39,1381)
Weight	5.09***	243.48***	0.95
Gain	(39,235)	(1,235)	(39,235)
Emergence	11.36***	-	-
Weight	(39,917)		

Summary from mixed-model ANOVA for the main effects of hemiclone and diet. Shown are F-statistics for each factor and corresponding degrees of freedom. Asterisks indicate levels of significance (*** P < 0.001).

**Table 2:** Patterns of genetic variation for all phenotypes.

	**Untransformed Data**^***a***^					Transformed Data			
**Trait**	**Environment**	**Mean**	**Heritability**^***b***^	**CV**_**A**_^***c***^	**CV**_**R**_^***d***^	**Mean**	**Heritability**	**CV**_**A**_	**CV**_**R**_

***Pre-Challenge Fecundity***	Yeast Limited	81.01	0.06 (0.03,0.13)	7.96	26.79	4.35	0.06 (0.04,0.13)	1.90	6.22
	Yeast Unlimited	203.50	0.17 (0.10,0.32)	6.47	13.73	5.30	0.15 (0.09,0.30)	1.24	2.78
***Bacterial load***	Yeast Limited	1.23 (x10^6^)	0.13 (0.06,0.38)	50.19	120.12	12.86	0.12 (0.06,0.38)	5.04	12.96
	Yeast Unlimited	1.20 (x10^6^)	0.08 (0.03,0.40)	33.89	110.82	12.93	0.14 (0.07,0.41)	5.18	12.70
***Day 9 Dry Weight***	Yeast Limited	539.60	0.21 (0.13,0.40)	5.47	10.28				
	Yeast Unlimited	612.82	0.26 (0.17,0.48)	5.41	8.85				
***Weight Gain***	Yeast Limited	147.12	0.30 (0.16,0.79)	19.07	27.30				
	Yeast Unlimited	220.31	0.40 (0.22,0.93)	14.55	17.27				
***Dry Weight At Emergence***	Larval	392.32	0.27 (0.17,0.47)	6.64	10.10				

^*a *^Data for fecundity and bacterial load was log-transformed prior to analysis in order improve the fit to normality. Values based on untransformed data are shown since estimates of the coefficient of variation on transformed data are difficult to interpret (Houle 1992).

^*b *^Heritability estimates and 95% confidence intervals (in parentheses) based on variance components from restricted maximum likelihood estimators.

^*c *^CV_A _– additive genetic coefficient of variation

^*d *^CV_R _– residual coefficient of variation. Based on restricted maximum likelihood estimators.

**Figure 1:**
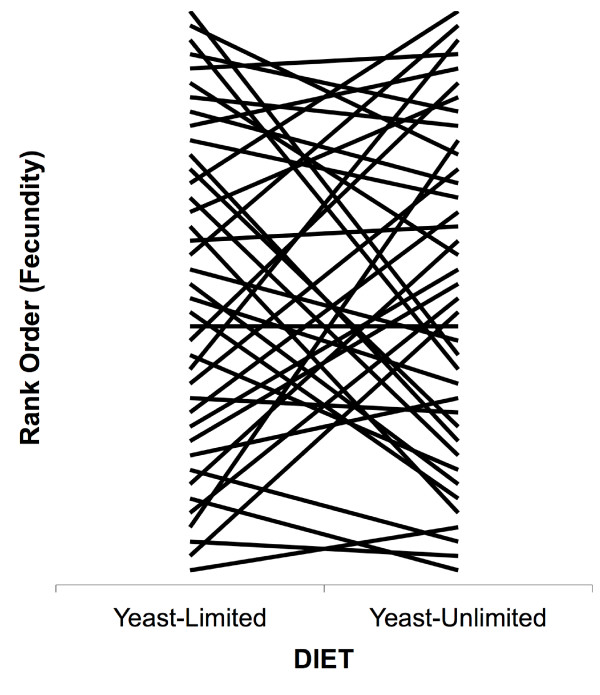
Genotype-by-environment interaction plot for the rank of hemiclone fecundity in yeast-limited and yeast-unlimited environments (ANOVA p < 0.0001; see Table 1 and text for details).

As expected, females in yeast-unlimited vials were more fecund than females in standard vials where yeast was limiting (Table 2). Additionally, there was a strong genotype-by-environment (GxE) interaction for fecundity (Fig. 1, Table 1). We estimated heritability and coefficients of variation for each environment separately. In the yeast-limited environment, the heritability of fecundity (*h*^*2 *^= 0.06) was just less than half that seen in the yeast-unlimited environment (*h*^*2 *^= 0.15). Estimates of the coefficient of additive variation for fecundity in each environment were very similar, while the residual variation (CV_R_) in the yeast-limited environment was almost twice that observed in the yeast-unlimited environment (Table 2).

While hemiclones varied in their ability to slow the growth of the experimental infection of *P. rettgeri*, there was no effect of diet and no diet × hemiclone line (GxE) interaction (Table 1). Estimates of the heritability and additive and residual coefficients of variation for immune defense were similar for both environments (Table 2).

Hemiclone lines varied in their dry weight at emergence, dry weight at day 9 post emergence and for the change in weight. Estimates of dry weight for females at day 9 post-emergence (and the related estimate of the change in weight over time) were based on uninjected females. The heritabilities of dry weight at emergence and dry weight at day 9 (in either food environment) were all very similar, ranging from 0.21 to 0.27 (Table 2). As expected, females provided the yeast-unlimited diet were heavier than females in yeast-limited vials. However, females in both environments gained weight over the course of the experiment, reinforcing our assumption that the yeast-limited environment represented limiting, but not starvation, conditions. Variation in weight gain also showed moderate levels of heritability (*h*^*2 *^= 0.21 in the yeast-limited environment and *h*^*2 *^= 0.40 in the yeast-unlimited environment). As with fecundity, there was evidence of a strong GxE interaction for female weight at day 9 post eclosion (Table 1).

For all of the phenotypes, estimates of CV_A _and CV_R _were similar for traits in the two environments. However, the residual variance tended to be higher in the yeast-limited environment for all of the phenotypes (Table 2). Among the phenotypes, both CV_A _and CV_R _were much higher for bacterial load.

**Figure 2:**
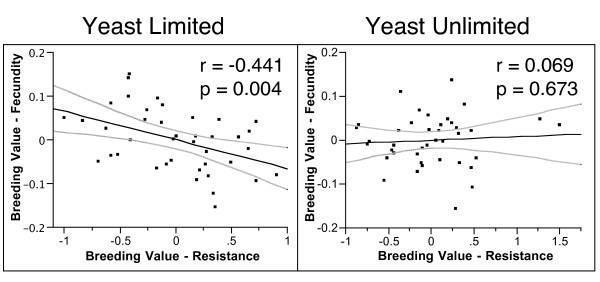
Genetic correlations between fecundity and resistance in yeast-limited and yeast-unlimited environments.

Genetic correlations were based on the Pearson product moment correlations of least-square hemiclone line means from the analyses described above. There was a strong, negative genetic correlation between fecundity and resistance in the yeast-limited environment (r = -0.441, P = 0.004). This correlation was absent in the yeast-unlimited environment (r = 0.069, P = 0.673). Using Fisher's z' transformation [32] we found these two correlation coefficients were significantly different from each other (P = 0.02; see Fig. 2).

No weight measure was significantly correlated with resistance (Table 3). Dry weight at day 9 was a significant predictor of fecundity in both environments (Yeast-limited environment: r = 0.337, p = 0.033; yeast-unlimited environment: r = 0.554, p = 0.0002).

**Table 3:** Genetic correlations among traits.

**Fecundity (Unlimited)**	0.357*	0.069	-0.043	0.554***	0.098	0.409**	-0.040	0.168
	**Fecundity (Limited)**	-0.159	-0.441**	0.438**	0.337*	0.299	0.189	0.171
		**Resistance (Unlimited)**	0.486**	0.056	-0.169	0.136	-0.062	-0.106
			**Resistance (Limited)**	-0.205	-0.270	-0.090	-0.129	-0.157
				**Day 9 Weight (Unlimited)**	0.641***	0.721***	0.347*	0.318*
					**Day 9 Weight (Limited)**	0.375*	0.670***	0.324*
						**Weight Gain (Unlimited)**	0.682***	-0.424**
							**Weight Gain (Limited)**	-0.482**
								**Emergence Weight**

* p < 0.05, ** p < 0.01, *** p < 0.001.

Weight at emergence did not correlate with subsequent fecundity in either environment (p > 0.25; Table 3). As expected, our various measures of weight were all highly correlated. Interestingly, there were strong negative genetic correlations between weight at emergence and weight gain in both environments (Yeast-limited environment: r = -0.482, p = 0.002; yeast-unlimited environment: r = -0.424, p = 0.006). Multiple regression of hemiclone line means for fecundity on resistance, with each of the different measures of weight entered as covariates, revealed that the negative correlation between fecundity and resistance seen in the yeast-limited environment was independent of size variation (Table 4).

**Table 4:** No effect of size variation on the trade-off between fecundity and resistance.

Covariate	Regression Coefficient (Covariate)	Regression Coefficient (Fecundity)
Emergence Weight	-0.002 (p = 0.578)	-2.863 (p = 0.007)
Day 9 Weight	-0.002 (p = 0.384)	-2.649 (p = 0.015)
Weight Gain	-0.001 (p = 0.753)	-2.899 (p = 0.007)

Shown are regression coefficients and associated p-values from multiple regressions of the line least-square means for fecundity and each size covariate onto resistance. Interactions between fecundity and each covariate were not significant (p > 0.4).

### Analysis of the cost of deployment

We analyzed the fecundity cost of immunological deployment using a contrast repeated measures ANOVA. The response design matrix (M-matrix) for the contrast repeated measures ANOVA compares the natural-log transformed fecundity in vial 3, prior to injections, with natural-log transformed fecundity in vials 4, 5, 6 and 7 separately. An initial analysis revealed significant interactions between diet and hemiclone line (Roy's Maximum Root, approximate F_39,691 _= 2.409, p < 0.0001) and a marginally significant interaction between diet and injection (Roy's Maximum Root approximate F_4,689 _= 2.435, p = 0.046). We therefore split the analysis between the two dietary manipulations (Table 5).

**Table 5:** Results from contrast repeated measures ANOVA for changes in fecundity after injection.

Yeast-Limited Diet							
	Full Model		Tests of Each Contrast Separately				

	d.f.	Roy's Max Root (approx. F)	d.f.	V3 vs. V4	V3 vs. V5	V3 vs. V6	V3 vs. V7

Hemiclone Line	39,343	3.122***	39,343	1.910**	2.375***	1.601*	1.693**
Injection	4,341	7.343***	2,343	5.638**	0.210	0.094	1.429
*HK vs. Sterile Needle*	4,340	1.362	1,343	0.051	0.122	0.001	2.807
*HK vs. Uninjected*	4,340	6.498***	1,343	9.041**	0.418	0.126	0.423
*St. Needle vs. Uninj*.	4,340	4.952***	1,343	7.811**	0.090	0.153	1.058
Hemiclone × Injection	78,343	1.337*	78,343	0.778	0.628	0.844	0.798
BLOCK	4,342	103.303***	3,343	14.430***	15.919 ***	11.041 ***	79.833 ***
Injector	4,343	16.612***	4,343	5.509***	4.006**	12.507***	7.039***
Number of Females	4,340	4.286 ***	1,343	6.203*	10.384**	15.008***	10.365**

**Yeast-Unlimited Diet**							

Hemiclone Line	39, 340	2.875***	39,340	1.727**	1.941**	0.653	1.615*
Injection	4,338	2.868*	2,340	4.882**	2.025	2.526	2.611
*HK vs. Sterile Needle*	4,337	2.466*	1,340	6.348*	3.284	5.045*	5.205*
*HK vs. Uninjected*	4,337	2.078	1,340	8.080**	2.591	1.052	1.505
*St. Needle vs. Uninj*.	4,337	0.629	1,340	0.110	0.048	1.435	1.068
Hemiclone × Injection	78,340	1.340*	78,340	0.685	0.834	1.159	1.034
BLOCK	4,339	72.312***	3,340	7.417***	4.992**	12.418***	43.740***
Injector	4,340	17.893***	4,340	11.535***	12.615***	3.556**	10.770***
Number of Females	4,337	37.617***	1,340	3.020	6.711*	100.065***	88.784***

The response matrix (M-Matrix) for the full model contrasts fecundity in Vial 3 (pre-injection fecundity) with each subsequent vial. Multivariate test statistics for the full model are based on approximate F-tests generated using Roy's Max Root.

* p < 0.05, ** p < 0.01, *** p < 0.001.

**Figure 3:**
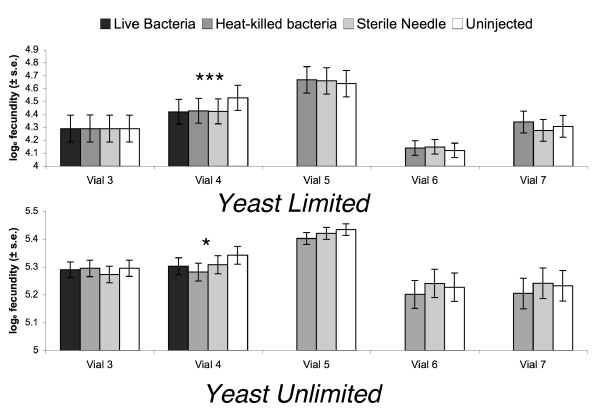
The effect of immune challenge on female fecundity. Stars indicate the significance of tests of the main effect of CHALLENGE from a model comparing pre-challenge fecundity (vial 3) to post-challenge fecundity (vials 4 – 7). See Tables 5 and 6 and the text for details on the statistical analysis. *** p < 0.001; * p < 0.05.

There was a strong effect of injection in the yeast-limited environment (approximate F_4,341 _= 7.343, p < 0.0001). This was mainly due to an approximate 10% decline in the fecundity of females in the first 28 hours after receiving an injection with either heat-killed bacteria or a sterile wound compared to the uninjected controls. In subsequent time periods the fecundity of females in the different injection treatments no longer differed (Fig. 3, Table 5).

Focusing on changes in fecundity in the first 28 hours after injection, we used a set of *a priori *orthogonal contrasts to test for differences between live-bacteria injected and heat-killed bacteria injected females (*C1*), between bacteria injected females and females injected with a sterile needle (*C2*) and between females receiving an injection of any sort and uninjected females (*C3*). In the yeast-limited environment, only *C3 *showed a decline in the fecundity of injected females compared to uninjected females (F_1,619 _= 11.756, P = 0.0006; Table 5) indicating significant costs of wounding that could not be distinguished from costs of immune system deployment or pathology associated with live bacteria.

As previously discussed, evolutionary costs of immunological deployment are revealed as a genotype-by-challenge interaction, indicating genetic variation for the physiological cost experienced. In the yeast-limited environment, this interaction was marginally significant (F_78,343 _= 1.337, p = 0.043). However, in the first 28 hours after injection, when the cost was realized, the interaction was not significant (Table 6). In order to examine the hypothesis more closely, we combined data across the three injection groups (live, heat-killed or sterile wound) and compared the fecundity of injected females to the fecundity of uninjected females in the first 28 hours after injection. Again, however, there was no indication of genetic variation in the cost experienced (LINE × INJECTION, F_39,384 _= 0.764, p = 0.848).

**Table 6:** Repeated measures ANOVA for changes in fecundity after injection.

Source	d.f	Yeast-Limited Diet	Yeast-Unlimited Diet
Hemiclone Line	39	1.677**	1.734**
Injection	3	4.104**	3.317*
*Orthogonal Contrasts*			
*1) Live vs. Heat-killed*	1	0.050	2.462
*2) Bacteria vs Sterile wound*	1	0.001	4.304*
*3) Injected vs Uninjected*	1	11.756***	4.147*
Hemiclone × Injection	117	0.898	0.907
BLOCK	3	19.071***	7.537***
Injector	4	5.962***	13.007***
Number of Females	1	6.479*	2.274

The analysis was split between the two diets. Tabular entries are F-tests for a model comparing the natural-log transformed fecundity in vial 3 (pre-injection fecundity) with the natural-log transformed fecundity in vial 4 (post-injection fecundity).

Injection treatment also had an effect in the yeast-unlimited environment (Roy's max root approximate F_4,338 _= 2.868, P = 0.023), although much less pronounced than that seen in the yeast-limited environment. However, unlike the situation in the yeast-limited environment, the effect was due primarily to the decline in fecundity in females receiving an injection with heat-killed bacteria, who showed a marginally significant decline compared to sterile-needle injected females (F_4,337 _= 2.466, P = 0.045) and a trend toward a significant decline compared to the uninjected females (F_4,337 _= 2.078, P = 0.083). There was no difference between sterile wounded females and uninjected females (F_4,337 _= 0.629, P = 0.642). Once again, the source of this effect was a difference apparent in the first day after injection, where females receiving an injection with heat-killed bacteria showed a strong decline in fecundity compared to both sterile wounded and uninjected females while sterile wounded and uninjected females were statistically indistinguishable (Table 5). Comparisons of pre-injection fecundity with fecundity at later time points did show a trend for females receiving an injection with heat-killed bacteria to have lower fecundity than both sterile wound and uninjected controls, but in no case was the main effect of injection significant (Table 5).

Fecundity in the yeast-unlimited environment in the first 28 hours after injection was analyzed in the same manner as described for the yeast-limited environment (see Table 6). Again fecundity varied on the basis of injection treatment (F_3,612 _= 3.317, P = 0.020). Examination of the *a priori *orthogonal contrasts showed no difference in the fecundity of females receiving injections with heat-killed bacteria versus injections with live bacteria (*C1*: F_1,612 _= 2.462, P = 0.117). However, there was a decline in the fecundity of bacteria-injected females compared to sterile-needle injected controls (*C2*: F_1,612 _= 4.304, P = 0.038). The effect seen in *C2 *makes the interpretation of the significance of the *C3 *comparison dubious. Post-hoc examination of the means for the different injection groups indicated that change in fecundity of females receiving an injection of bacteria (live or heat-killed) was lower than for uninjected females (F_1,612 _= 7.068, P = 0.008) and that there was no difference between uninjected and sterile needle injected females (F_1,612 _= 0.223, P = 0.637).

Once again, the line by injection interaction was marginally significant for the full model, but was not significant for the analysis of changes in fecundity in the first 28 hours after injection (Table 6). To examine this hypothesis more closely we combined data for bacteria-injected females (either with live or heat-killed bacteria) and compared their fecundity to the combined data of the two controls (injected and sterile wound). As in the yeast-limited environment, lines did not differ in the costs experienced (LINE × INJECTION (bacteria vs. control): F_39,380 _= 0.817, p = 0.777).

**Figure 4:**
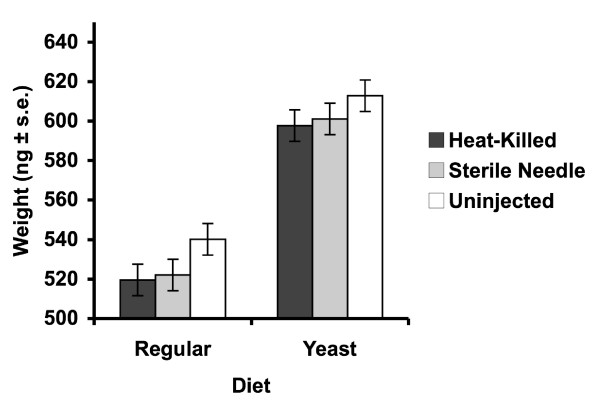
The effect of immune challenge on female dry weight.

In terms of female dry weight at the end of the experiment, females receiving an injection (either with heat-killed bacteria or a sterile wound) had a lower weight than the uninjected controls in both environments (Fig. 4, Table 7). The line × injection interaction, indicative of an evolutionary cost of deployment, was significant in the yeast-unlimited environment, and trended towards significance in the yeast-limited environment (Table 7).

**Table 7:** Summary from mixed-model ANOVA for dry weight on Day 9 (the end of the experiment).

**Source**	**d.f.**	**Yeast-Limited Diet**	**Yeast-Unlimited Diet**
Hemiclone Line	39	12.951 (p < 0.0001)	15.960 (p < 0.0001)
Injection	2	27.896 (p < 0.0001)	14.693 (p < 0.0001)
*HK vs. Sterile Wound*	1	0.596 (p = 0.440)	1.135 (0.287)
*HK vs. Uninjected*	1	46.294 (p < 0.0001)	26.282 (p < 0.0001)
*Sterile Wound vs. Uninjected*	1	36.478 (p < 0.0001)	16.266 (p < 0.0001)
Line × Injection	78	1.236 (p = 0.082)	1.341 (p = 0.027)
BLOCK (random)	3	---	---
Injector (random)	4	---	---

Shown are F-statistics for each factor with corresponding p-values in parentheses.

## Discussion

### The evolutionary cost of immunological maintenance

The experimental manipulation of yeast availability had a predictably large effect on female fecundity, with females in the yeast-unlimited environment (${\bar{X}}_{fecundity}$ = 203.5 offspring) having over 2.5 times more offspring than females in the yeast-limited environment (${\bar{X}}_{fecundity}$ = 81.0 offspring). Previous studies in Drosophila and other insect species have repeatedly shown an association between fecundity and the nutritional status of females [33,34]. Females in yeast-limited vials were not starving as evidenced by the gain in dry weight from emergence to the end of the experiment 9 days later (Table 2). Of course weight gain was also observed in females on the yeast-unlimited diet, and by the end of the experiment these females weighed 13.6% more than females on the yeast-limited diet.

The dietary manipulation leads to strong genotype-by-environment interactions for both fecundity and dry weight at day 9 (Table 1). The inter-environmental genetic correlation for trait values across the two environments indicates that a shared set of genes contributes to variation in these traits in both environments (Fecundity: r = 0.357, P = 0.024; day 9 weight: r = 0.641, P < 0.0001). Nevertheless, the significant genotype-by-environment interaction for both of these traits suggests fundamental differences in genetic architecture across this environmental gradient. Such an effect could arise if, for example, polymorphism in genes involved in resource acquisition had different effects on fecundity in the two environments, (perhaps as a consequence of costs of acquisition [27]). Alternatively, the hormonal response to different food availabilities [35] could lead to differences in the set of genes expressed in the two environments, thereby unmasking independent sources of genetic variation.

Unlike fecundity and adult dry weight, there was no discernable effect of the environmental manipulation on immune function. Estimates of the number of bacteria recovered were nearly identical (Table 2) and there was no genotype-by-environment interaction (Table 1, F_39,518 _= 1.277, P = 0.127). It is surprising that diet did not affect immune function given that a previous study, with a similar manipulation of food availability, found that females with *ad libitum *access to dietary yeast had dramatically improved immunity compared to females on a yeast-limited diet [36].

This study differed in 3 ways that could potentially cause the disparity in outcomes. First, the studies used flies from different populations. However, given that there is little genetic differentiation among North American populations of *D. melanogaster *[37] this does not seem a satisfactory explanation for the dramatically different outcomes. A second difference is that McKean and Nunney (2005) looked at the clearance of non-pathogenic bacteria, *E. coli*, rather than the ability to slow the growth of a pathogenic bacterium. This also seems wanting as an explanation as the slower clearance of *E*. coli for females on the yeast-limited diet was mirrored by a more rapid death from an experimental infection with pathogenic *Pseudomonas aeruginosa *[36].

A third explanation, and the one that seems most likely, is that there are differences in the conditions actually represented by the environmental manipulation performed. The 'standard' yeast-limiting vials used in this study were made with agar-dextrose-yeast media while in the study of McKean and Nunney (2005) standard, yeast-limiting vials were made with agar-cornmeal-molasses. In both studies it seems likely that the yeast-supplemented vials represented *ad lib *food conditions. However, it appears that the low food environment in the study of McKean and Nunney (2005) may have represented a greater limitation for females than in the present study. In support of this hypothesis, the fecundity of females in yeast-unlimited condition in McKean and Nunney (2005) was 6.7 times that of the yeast-limiting condition, compared to only a 2.5 fold increase seen in the present study. The results of McKean and Nunney (2005), utilizing the agar-cornmeal-molasses food, have recently been replicated (Bedhomme et al. in prep.) suggesting that the effect of food availability on patterns of immune function is a threshold trait, and that past a certain level of food availability further improvements in immune function do not occur.

Our analysis revealed heritable variation for each of the phenotypes assayed. These heritability estimates represent the upper bound of estimates of the narrow sense heritability [38]. There are three potential sources of confounding variation that could inflate our estimate of the additive genetic variance. The first two arise as a consequence of how we sampled test females from the hemiclone families and the third is particular to using the clone-generator system for estimating the breeding values.

Due to the very large number of flies needed for this experiment (a total of 11,360 test females), we did not attempt to precisely control the larval density or to keep track of the source of maternal chromosomes. It seems unlikely that variation in larval density would inflate our estimate of the additive variance. The numbers of females laying eggs in the collection vials was quite low and because the females in these vials were fully wild-type, variation among vials in the density of eggs laid is expected to be random with respect to hemiclone genotype. It also seems unlikely that our estimate of the additive variance is inflated by the confounding of non-additive genetic variance arising from a pervasive sampling of full sibs, in violation of our assumption that test females were almost exclusively composed of half-sibs. The test females used within each block of the experiment are the offspring of 75 randomly sampled females from our base population (15 females × 5 separate rearing vials). Assuming an equal contribution of daughters from each female, the probability that two randomly sampled test females within a particular test vial of 5 females are not full sibs is 0.998. Therefore, it is not likely that estimates of the additive variance are inflated due to violation of the half-sib assumption.

A third source of deviation from more standard quantitative genetic designs is unique to the clone-generator system itself [38]. The experimental design used here gives an estimate of the *breeding value of a gamete*, while a half-sib design provides estimates based on the breeding value of an individual [39,40]. For a species like *D. melanogaster*, where there is no recombination in males, the breeding value of a gamete could differ from the breeding value of an individual male if there are strong epistatic interactions between allelic variants at loci on different chromosomes [38]. However, since only 3 chromosomes make up almost 99% of the genic content in *D. melanogaster*, it is unlikely that such non-additive effects would inflate the additive variance. Furthermore, the present design is very similar to other quantitative genetic designs in *D. melanogaster *utilizing balancer chromosomes [i.e., the North Carolina II breeding design; [40]] or crossing designs (such as diallels) using highly inbred lines.

Our estimates for the heritability of fecundity, immunity and dry weight in the two environmental conditions (Table 2) are consistent with expectations from previous studies of life history traits [41-43]. Life history traits tend to exhibit lower heritability than morphological traits presumably because life history traits experience strong selection [16,41,42,44] although there are other explanations [43,45].

Houle [43] has argued that a more appropriate measure of genetic variation is the mean standardized additive variance, the coefficient of additive variation, CV_A_, rather than the heritability. In general, the low heritability of life history traits, such as fecundity, appears to arise from a greater residual variation rather than an absolute reduction in the additive genetic variance. Such an effect seems to explain why we see such a low estimate for the heritability of fecundity in the yeast-limited environment (*h*^*2 *^= 0.06) compared to the yeast-unlimited environment (*h*^*2 *^= 0.15); a comparison of CV_A _shows they are similar in the two environments, however, the CV_R _in the yeast-limited environment is twice that seen in the yeast-unlimited environment (Table 2).

Our estimates for the heritability of the ability to slow the growth of *P. rettgeri *(Table 2) are much lower than those reported for immune-related traits in other insect species. For example, in the field cricket, *Teleogryllus oceanicus*, the heritability of encapsulation response (*h*^*2 *^= 0.48 ± 0.06) and hemocyte load (*h*^*2 *^= 0.74 ± 0.06) are more than three times as high as our estimates of resistance to *P. rettgeri *in the yeast-limited (*h*^*2 *^= 0.12) and yeast-unlimited environment (*h*^*2 *^= 0.14). Similar observations of very high levels of heritability have been reported for the caterpillar *Spodoptera littoralis *(phenoloxidase (PO) activity: *h*^*2 *^= 0.69 ± 0.07; encapsulation: *h*^*2 *^= 0.62 ± 0.14 [46]), in the Egyptian cotton leafworm (cuticular melanization: *h*^*2 *^= 0.36 ± 0.08; PO activity: *h*^*2 *^= 0.65 ± 0.11; antibacterial activity: *h*^*2 *^= 0.63 ± 0.11; haemocyte density: *h*^*2 *^= 0.36 ± 0.08; [47]), and in the yellow dung fly (PO activity: *h*^*2 *^= 0.69 ± 0.48 [48]).

The exact origin of such profound differences in the magnitude of the heritability estimates is unclear. One possibility is that functional measures of the effectiveness of an immune response may be quite different than measures of single effectors or components of the immune response [49]. For example, the ability to slow the growth of a pathogenic bacterial population is likely a multifaceted interaction between various mechanisms of host defense and particular virulence mechanisms possessed by the bacteria. If interactions among the various components of the immune response contribute a large amount of epistatic variance, or if environmental variation affecting each component of the response combines to increase the total variance in the functional outcome, then this could reduce the heritability of functional response even though each component may show high levels of heritability. Consistent with this hypothesis, the heritability of the melanization response to sephadex beads in the mosquito *Anopholes gambiae *was much greater than observed for resistance to *E. coli *[50].

Evolutionary costs of immunological maintenance are revealed as negative genetic correlations between fitness components in the absence of infection and immune system function [10,51]. Our results clearly indicate the presence of such costs, but that their expression is condition dependent. In a nutritional environment in which food is limiting, there was a strong negative genetic correlation between female fecundity and resistance to the bacterial infection (r = -0.441, P = 0.004). However, as the nutritional environment improved, this trade-off was no longer present (r = 0.069, P = 0.673). In fact, the two correlation coefficients are significantly different from each other (z-test, p = 0.02) indicating a significant genotype-by-environment interaction for the genetic correlation [31]. Such results indicate that predicted evolutionary trajectories based on estimates obtained in only one environment may be highly misleading. In this case, all else being equal, correlated effects on fecundity may slow the short-term response to selection for resistance only when food is limiting, while if food were not limiting, these traits would be predicted to evolve independently.

The effect of environmental variation on evolutionary costs of immunological maintenance has also been observed in lines of *D. melanogaster *selected for increased resistance to the larval parasitoids *Asobara tabida *or *Leptipolina boulardi *[2,4]. These results illustrate the importance of considering multiple ecologically relevant environments when estimating patterns of genetic variance and covariance of life history traits, including immune function [29-31,52].

### The costs of immunological deployment

An immunological cost of deployment is recognized as a reduction in fitness as a consequence of immune system activation [10,15,53]. The analysis of immunological deployment costs can focus on physiological costs, (i.e., the immediate cost of immune system activation), or evolutionary (genetic) costs, (i.e., whether there is genetic variation for the physiological cost experienced). There is a growing literature on studies examining the physiological costs of immune system activation [10,13-15]. Indeed, the absence of deployment costs would be troubling, raising the question of why defense mechanisms are inducible and not simply constitutively expressed [22]. The evolutionary costs of immunological deployment have not been well studied.

The experimental design used here allows us to examine both the physiological and evolutionary costs of immunological deployment. Our results suggest the presence of short-term physiological costs of deployment (Fig. 3 and 4, Tables 5, 6, 7). The effect of the different injections on fecundity and female dry weight differed between the two environments, with a slight exaggeration of costs when females were under yeast-limited environmental conditions.

Females in the yeast-limited environment were less fecund in the first 28 hours after injection, but fecundity returned to uninjected levels by 48 hours after infection (Fig. 3). The decline in fecundity of females receiving an injection of bacteria (either live or heat-killed) was similar to the decline observed for sterile-wound females, meaning that costs of wounding could not be distinguished from costs associated with the response to bacterial challenge (Fig. 3). The wound response in *D. melanogaster *involves the production of antimicrobial peptides and the activation of enzyme cascades involved in wound repair. These induced responses to wounding could contribute to the observed fecundity cost. The cost could also arise as a consequence of physical damage. However, it would seem that physical damage would have longer-lasting effects on fecundity instead of the transitory effect observed here.

Our results differ dramatically from those reported by Zerofsky et al. [54]. In their study females were injected with a mixture of heat killed *Micrococcus luteus *(a Gram-positive bacterium) and *Escherichia coli *(a Gram-negative bacterium). The fecundity of these females was compared to sterile media injected controls. Wild-type, immune intact, females experienced a significant and long lasting decline in fecundity [54].

We suggest two hypotheses to explain the disparity in 1) distinguishing changes in fecundity in sterile-wound and bacteria injected females and 2) the lack of a long-term fecundity cost in this experiment. First, there was a tremendous difference in the amount of bacteria introduced to females in the 2 experiments. In the study of Zerofsky et al. [54], overnight cultures of *M. luteus *and *E. coli *were mixed, heat-killed, centrifuged and then the needle dipped into this highly concentrated pellet. We used solutions containing heat-killed bacteria diluted to an OD_610 _≈ 0.6, or live bacteria at a slightly lower concentration (OD_610 _≈ 0.2). The apparent active and rapid down-regulation of immune responses [55,56] may mean that significant deployment costs are only observed following persistent immune system induction. Second, the combined activation of both the Toll and imd pathways (by mixing the Gram-positive *M. luteus *and Gram-negative *E. coli*) could produce longer-lasting costs. Consistent with this is the observation that the E38, *relish *mutants (deficient in the production of *imd*-regulated antimicrobial peptides) did not experience a cost of deployment [54] even though the Toll pathway, presumably activated in response to the *M. luteus*, was intact.

We also observed a deployment cost in the yeast-unlimited environment, and as with the yeast-limited environment, this cost was short term, with fecundity returning to the level of uninjected controls within 48 hours. The unlimited access to dietary yeast affected both the magnitude and the type of cost experienced. First, the 5% decline in fecundity of bacteria-injected (either live or heat-killed) females in the first 28 hours after infection was less than the 11% decline observed for similarly challenged females on the yeast-limited diet. Second, in the yeast-unlimited environment a cost of immune system activation could be distinguished from a simple cost of wounding (Tables 5 and 6, Fig. 3).

There are other examples of food availability affecting deployment costs and also the cost of parasitism itself. For example, in the bumble bee, *Bombus terrestris*, the acceleration of mortality following challenges with LPS and synthetic beads was only seen when bees were starved subsequent to the challenge and not in bees kept on a normal diet [26]. Likewise, in *Spodoptera littoralis *caterpillars immune function declined, but the cost of nucleopolyhedrovirus increased, as a consequence of manipulations of dietary protein levels [57]. However, *ad libitum *access to food does not appear to allow for complete mitigation of deployment costs as this and another study [58] have demonstrated.

A physiological cost of deployment was also reflected in the decline in dry weight of females injected with heat-killed bacteria or given a sterile wound. This decline in weight was seen in both the yeast-limited environment, where there was a 3.7% decline in dry weight, and in the yeast-unlimited environment where females exhibited a 2.3% decline in weight compared to uninjected controls (Table 7, Fig. 4). This observation is interesting in light of work demonstrating a type of 'wasting' in flies following infection with *Mycobacterium marinum *[59]. In that study, infection promoted a progressive loss of energy reserves and hyperglycemia as a consequence of hyperactivation of the transcription factor FOXO. Dionne et al. [59] argued that the appearance of wasting was an unintended byproduct of a massive reallocation of energy reserves towards immune function. An alternative hypothesis is that the pathology arises as a consequence of the fitness promoting activities of the pathogen [60]. Our results, indicating a decline in dry weight in females receiving either a sterile wound or heat-killed bacteria, are consistent with the energy reallocation hypothesis, although the role of injection-induced anorexia cannot be ruled out.

Lastly, we did not find overwhelming evidence of evolutionary costs of immunological deployment. For fecundity, there were marginally significant hemiclone line by injection interactions for the full model in the contrast MANOVA (within each environment, Table 5). However, when comparing changes in fecundity in the first 28 hours after the challenge (the contrast of Vial 3 and Vial 4 when costs were apparent) the interaction was not significant (Tables 5 and 6). Dry weight did show a significant hemiclone line × injection interaction in the yeast-unlimited environment (p = 0.027), and a marginally significant interaction in the yeast-limited environment (p = 0.082).

Only one previous study has examined the extent of genetic variation for the cost of immunological deployment, however in that study costs of immunity could not be distinguished from costs of parasitism [25]. Our inability to demonstrate evolutionary costs of immunological deployment may reflect a lack of statistical power, however, the analysis suggests a rather small bound on the magnitude of evolutionary costs compared to the GxE interactions observed for fecundity and for the genetic correlation between fecundity and resistance. One explanation is that if immune responses are tightly regulated, as appears to be the case [56], the extent of immune induction represented by the challenges represented in this study may be so rapidly down-regulated that genetic variation in this regulation is relatively unimportant. Thus, a more substantial immunological challenge, or a series of smaller challenges, resulting in prolonged activation of the immune response, may uncover underlying genetic variation for deployment costs. Furthermore, subtle evolutionary costs of deployment beyond the power of the present study could still be of evolutionary importance in the long term. At the very least, genetic variation for deployment costs must have existed in the past and it will be interesting in future research to explore whether such variation can be unmasked and the potential interaction between the evolutionary costs of deployment and the closely related phenomenon of tolerance [61].

## Conclusion

In summary, the results presented here suggest that fitness costs of immunological maintenance and deployment may constrain populations from achieving high levels of resistance, especially in food-limited environments. In fact, in an environment in which individuals have *ad libitum *access to dietary yeast, our results suggest that selection for improved resistance to *P. rettgeri *could proceed independent of effects on fecundity, at least in the short term. Our results emphasize the importance of examining costs in variable environments, and that food availability in particular is an important factor affecting patterns of genetic variation and correlation among fitness traits including immune system function.

## Methods

### Base population

We established the population of *Drosophila melanogaster *used in this study from 139 isofemale lines captured at the Little Tree Apple Orchard (Newfield, NY) in the summer of 2004. The population was maintained as isofemale lines until August 2005, when we created a large outbred population from equal numbers of mated females from each of the lines. We maintained this population for 6 generations prior to isolating the hemiclones used in the experiment. In general, adaptation to laboratory conditions occurs very rapidly [62-64]. Thus, it is unlikely that genotype-by-environment interactions, characterizing the early adaptation to lab conditions, affected our estimates of patterns of genetic variance and covariance.

During the first 5 generations of outbreeding, we placed 15 females and 15 males in each of 250 vials (N = 7500). After 24 hours we removed the adults from the vials leaving only the eggs. Twelve days later, we collected the next generation of adults and mixed evenly among vials. In the generation prior to establishing the hemiclones, we randomly assigned 6 males and 6 females from each of the 250 vials to one of 100 vials (again maintaining 15 males and 15 females per vial). We maintained the population in a similar manner (15 males and 15 females in each of 100 vials) during cytogenetic cloning and amplification.

### Cytogenetic cloning and creation of hemiclone line families

**Figure 5:**
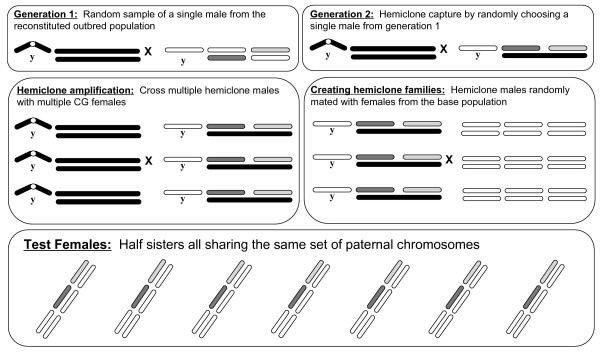
Crossing scheme for the generation of paternal half sib sisters using 'clone generator' (CG) females. CG-females carry a compound × chromosome [C(1)DX, *y*, *f*], a Y chromosome, and are homozygous for a translocation of chromosomes 2 and 3 [T(2;3) *rdg*C *st in ri p*^*P *^*bw*]. The translocated chromosomes are represented as a solid black bar because offspring are viable only if both chromosomes are inherited. The compound X ensures that during hemiclone capture and amplification, males receive their wild-type X chromosome from their father, and the absence of recombination in males ensures there is no mixing of wild-type and CG autosomes.

We isolated a total of 100 hemiclones from our sample population and from this sample randomly chose 40 for use in this study. The sampling of hemiclones (cytogenetic cloning) has been previously described [38,65]. The process is essentially an amplification of a randomly sampled X-chromosome-carrying gamete representing an intact haploid set of genes found on chromosomes I, II and III, but excluding the 'dot' chromosome IV (which represents less than 0.5% of the genic content in the *D. melanogaster *genome). Cytogenetic cloning relies on the absence of recombination in males and the use of so-called 'clone generator' (CG) females. The process of hemiclone isolation, amplification, and the creation of hemiclone families for quantitative genetic analysis is described in Figure 5. In the first generation, we crossed single, randomly sampled, wild-type males from the base population with 5 virgin CG-females. The resulting male offspring carry maternally derived CG chromosomes, but are variable due to the independent assortment of paternal chromosomes. The male offspring of the second generation cross between a single F1 male with 5 CG females all carry identical copies of paternally derived wild-type chromosomes 1, 2 and 3, thus representing hemiclone capture. Subsequent generations of matings between multiple hemiclone males with multiple CG-females allows for hemiclone amplification (Fig. 5).

In this study we examined immunological costs in females only. Experimental females were founded by crossing hemiclone males randomly to females from the base population. For each of the 40 hemiclone lines used in the study, 5 replicate vials of 10 hemiclone males combined with 15 virgin females from the base population were established and transferred every 24 hours for 2 days. In this crossing design it is expected that 1/2 of fertilized eggs will be inviable, thus these conditions will result in relatively low larval densities. Furthermore, variation in larval density among collection vials due to variation in female fecundity should be random with respect to the hemiclones and thus should not act to confound subsequent phenotypic assays.

We mixed the newly emerged virgin females from all 10 vials before placing them randomly in experimental vials (see below). These fully wild-type females share the same set of paternal chromosomes (derived from the hemiclone male) and a random set of maternal chromosomes and mitochondria. Assuming that most of the sampled offspring are not full sibs, these 'hemiclone-families' are composed of half-sisters that, because they share the same paternal set of chromosomes, have a coefficient of relatedness of 0.5. For any phenotype of interest, the deviation of the hemiclone family mean from the population mean phenotype gives a direct estimate of the breeding value of a gamete. The correlation of breeding values for different traits, or for the same trait expressed in different environments, is the broad-sense genetic correlation and the heritability can be estimated from the among family variance, which provides an upper bound to the additive genetic variance, *V*_*A *_[38].

### Experimental design

**Figure 6:**
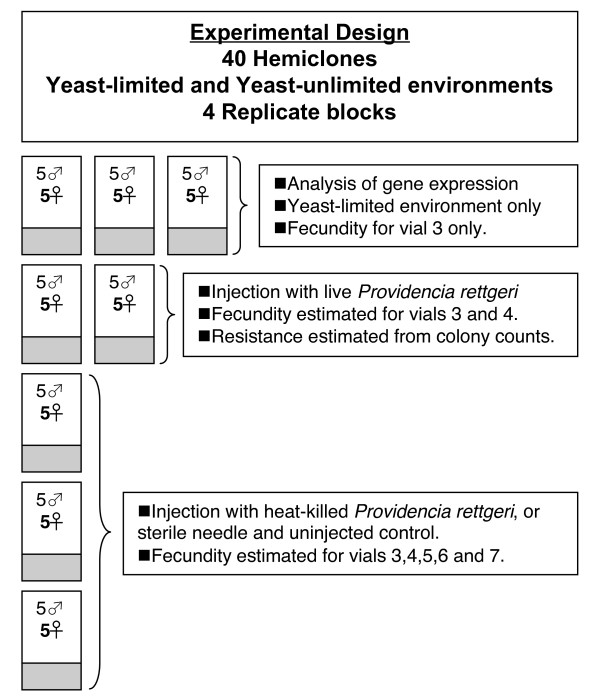
Outline of the experimental design. Eight standard (yeast-limited) vials and 5 yeast-supplemented vials were set up for each hemiclone family, consisting of 5 test females and 5 randomly sampled wild-type males. Females were collected at emergence (vial 1), transferred to a new vial 2 days later (vial 2) and then into vial 3 after 2 more days. Counts of emerging offspring were conducted for vials 3 – 7, with females transferred every 24 hours between these vials. Injections were carried out during the transfer of females from vial 3 to vial 4. Females receiving an injection of live *Providencia *rettgeri were homogenized 28 hours after injection and resistance quantified from bacterial counts. For each hemiclone, two vials were set up in each environment to evaluate resistance. Fecundity in vial 3 was used to evaluate the cost of immunological maintenance. Deployment costs were evaluated by comparing pre-challenge fecundity (vial 3) to post-challenge fecundity (vials 4–7). Dry mass was estimated for females collected at emergence and for females collected at the end of the experiment (exiting vial 7). Females from the three vials used in an analysis of constitutive gene expression were frozen at the time of injections. Data on gene expression is being published elsewhere. See text for details on the analyses.

Our goals were to measure the evolutionary costs of immunological maintenance and deployment in food-limited and food-unlimited environments. An outline of the experimental design is shown in Figure 6. We measured maintenance costs as the genetic correlation between resistance to an experimental bacterial infection of *Providencia rettgeri *and fecundity in the absence of infection. Deployment costs were evaluated as the decline in female fecundity following injections with either live or heat killed *P. rettgeri *compared with both sterile needle injected and uninjected controls. The design described below, and shown in Figure 6, is for one replicate block of the experiment. The entire experiment was composed of 4 replicate blocks, each representing an independent sample of females from the base population used to establish the hemiclone families.

We manipulated adult food availability by adding dietary yeast to 'standard' vials. Standard vials were made with equal amounts, by weight, of glucose and yeast and these represent yeast-limited, but not starvation conditions. Yeast-supplemented vials were made by placing 40 mg of yeast suspended in 50 *μ*l of water onto the surface of the food in standard vials. The vials were then allowed to dry for 2 – 3 days prior to use in the experiment. This amount of yeast was not exhausted during the 24 hours the females were in vials, thus this manipulation represents an *ad libitum *amount of food.

We collected experimental, virgin females as described above and placed 5 per vial in 8 standard and 5 yeast-supplemented vials (N = 520 vials per replicate block). We froze females from the 3 extra standard vials on the day of injections to examine patterns of variation in immune system gene expression, the results of which will be published elsewhere. We collected an additional 6 experimental females and froze them for subsequent analysis of female dry weight at emergence. On day 2, we transferred the females to a new vial containing 5 males randomly sampled from the base population. On day 4, we transferred the flies to a new vial (vial 3) and discarded vial 2. On the afternoon of day 5, we injected the flies (see below) and placed them in vial 4. We then transferred the flies every 24 hours for 3 more days (representing vials 5, 6 and 7). On day 9, we transferred the flies out of vial 7 and into microcentrifuge tubes and flash-froze them for subsequent analysis of female dry weight at the end of the experiment. We counted the number of emerging offspring for each of vials 3 – 7 representing our estimate of female fecundity. We determined the dry weight of females at emergence and on day 9 by drying the flies in a drying oven for 24 hours, and then weighing each individual to the nearest 0.001 *μ*g using a Sartorius CP2P microbalance (Data Weighing Systems, Elk Grove, IL).

On the day of infections, we injected the females with either live *Providencia rettgeri*, or heat-killed *P. rettgeri*. Controls for the analysis of deployment costs included both sterile-wounded and uninjected females. *P. rettgeri *is a Gram-negative bacterium in the family Enterobacteriaceae. *Providencia *species have been isolated from a number of different insects, including Drosophila [66]. B. Lazzaro isolated the *P. rettgeri *strain used in this experiment from the hemolymph of a wild-caught *D. melanogaster *collected near State College, Pennsylvania, USA. This strain is pathogenic to *Drosophila*, meaning that it is able to grow rapidly and cause fly death when introduced into the hemocoel.

On the evening prior to infections, bacterial cultures were initiated in sterile LB and allowed to grow overnight at 30 C. We diluted the resulting cultures to an optical density of A_610 _= 0.6 (for injections with heat-killed bacteria) or A_610 _= 0.2 (for injections with live bacteria). We heat-killed the bacteria by placing a culture at 65 C for 45 minutes prior to injections. To test the effectiveness of the heat killing process, we plated a 50 *μ*l sample of each of these cultures and in no case were live bacteria observed. We injected the flies by piercing their thorax with a 0.1 mm minutien pin (Fine Science Tools, Foster City, CA) dipped into either 1) a liquid culture of live *P. rettgeri*, 2) a liquid culture of heat-killed *P. rettgeri *or 3) sterile LB. We used separate needles for each injection treatment. Uninjected and injected females were handled in a similar manner with respect to the timing of CO_2 _anesthetization.

For females receiving an infection of live bacteria, we estimated the bacterial load 28 hours after infection by homogenizing 3 females in 500 *μ*l of sterile LB and plating 50 *μ*l of this homogenate on LB plates with an Autoplate 4000 spiral plater (Spiral Biotech, Bethesda, MD). The plates grew overnight at room temperature and we then counted the number of colony forming units (CFU) using the Q-Count detection system (Spiral Biotech, Bethesda, MD). We plated a total of 8 plates for each hemiclone line in each environment.

### Analysis of the evolutionary cost of immunological maintenance

As discussed above, a negative genetic correlation between immunological performance, assayed as the ability to slow the growth of pathogenic *P. rettgeri*, and fecundity in the absence of infection is indicative of an evolutionary cost of immunological maintenance. We estimated genetic correlations as the parametric (Pearson product-moment) correlations of least-square hemiclone line means for our phenotypes from mixed-model ANOVAs outlined below. The analysis of fecundity in the absence of an immune response is based on counts of emerging offspring from vial 3. This represents counts of emerging offspring from 8 vials in the yeast-limited diet and 5 vials in the yeast-unlimited diet within each of the 4 replicate blocks (n = 32 for the yeast-limited diet and n = 20 for the yeast-unlimited diet). Counts of emerging offspring were natural-log transformed in order to improve the fit to normality. We performed a mixed-model analysis of variance (ANOVA) on the natural log transformed counts of emerging offspring using the following model:

(1) *Y*_*ijkl *_= *μ *+ *L*_*i *_+ *D*_*j *_+ (*LD*)_*ij *_+ *b*_*k *_+ *ε*_*ijkl*_

where *μ *is the grand mean, *L*_*i *_is the fixed effect of the *i*^*th *^hemiclone LINE (*i *= 1,2,...,40), *D*_*j *_is the fixed effect of the *j*^*th *^DIET (*j *= yeast-limited or yeast-unlimited), (*LD*)_*ij *_is the LINE × DIET (genotype-by-environment) interaction, *b*_*k *_is the random effect of the *k*^th ^BLOCK (*k *= 1,2,3,4) and *ε*_*ijkl *_is the residual variance. Estimation of the LINE least-square means and measures of variation (heritability and the additive and residual coefficients of variation) for fecundity in the absence of infection were estimated for each environment separately by entering the effect of LINE as a random factor into the model. Breeding values, used to establish genetic correlations among traits, were calculated as the deviation of the LINE least-square means from the population mean.

We analyzed resistance to *P. rettgeri *infection using a mixed-model analysis of variance (ANOVA) on the natural log transformed counts of *P. rettgeri *colonies with the following model:

(2) *Y*_*ijklm *_= *μ *+ *L*_*i *_+ *D*_*j *_+ *(LD)*_*ij *_+ *i*_*k *_+ *b*_*l *_+ *ε*_*ijklm*_

where *L*_*i *_is the fixed effect of the *i*^th ^hemiclone LINE (*i *= 1,2,...,40), *D*_*j *_is the fixed effect of the *j*^th ^DIET (yeast-limited or yeast-unlimited), (*LD*)_*ij *_is the LINE × DIET (genotype-by-environment) interaction, *i*_*k *_is the random effect of the *k*^th ^INJECTOR (*k *= 1,2,3,4,5), *b*_*l *_is the random effect of the *l*^th ^BLOCK (*l *= 1,2,3,4) and *ε*_*ijklm *_is the residual variance. Colony counts were natural-log transformed in order to improve the fit to normality. Again, we calculated the hemiclone LINE least-square means and measures of variation separately for each environment. For the analysis of genetic correlations, we calculated the breeding value for resistance by subtracting the LINE least-square mean from the population mean. Thus positive values indicate hemiclone lines in which fewer bacteria were recovered (i.e., hemiclone lines better able to slow the growth of the bacteria).

We also collected data on female weight. Weight data included weight at emergence, weight at day 9, and from these two measures we could also calculate the change in weight. We evaluated among hemiclone line variation in emergence weight using the following mixed ANOVA model:

(3) *Y*_*ijklm *_= *μ *+ *L*_*i *_+ *b*_*j *_+ *ε*_*ijl*_

Where *μ *is the grand mean, *L*_*i *_is the fixed effect of the *i*^th ^hemiclone LINE (*i *= 1,2,...,40), *b*_*j *_is the random effect of the *j*^th ^BLOCK (*j *= 1,2,3,4) and *ε*_*ijkl *_is the residual variance.

At the end of the experiment we weighed uninjected females along with females that had received either heat-killed bacteria or a sterile wound. We analyzed day 9 dry weights using the following model:

(4) *Y*_*ijklmn *_= *μ *+ *L*_*i *_+ *D*_*j *_+ (*LD*)_*ij *_+ *C*_*k *_+ (*LC*)_*ik *_+ (*DC*)_*jk *_+ (*LDC*)_*ijk *_+ *i*_*l *_+ *b*_*m *_+ *ε*_*ijklmn*_

where *μ *is the grand mean, *L*_*i *_is the fixed effect of the *i*^th ^hemiclone LINE (*i *= 1,2,...,40), *D*_*j *_is the fixed effect of the *j*^th ^DIET (*j *= yeast-limited or yeast-unlimited), *C*_*k *_is the fixed effect of the *k*^th ^immune CHALLENGE (heat-killed, sterile needle, or uninjected), *b*_*k *_is the random effect of the *k*^th ^BLOCK (*k *= 1,2,3,4), *i*_*l *_is the random effect of the *l*^th ^INFECTOR (*l *= 1,2,3,4,5) and *ε*_*ijkl *_is the residual variance. We included all 2-way interactions and the 3-way interaction in the model. We used a reduced model to estimate the heritability of dry weight at day 9, including only females in the uninjected group, split between the two diets. We analyzed weight gain during the experiment by first subtracting the emergence weight from the weight at day 9 of uninjected individuals within each diet and then applying the following ANOVA model:

(5) *Y*_*ijk *_= *μ *+ *L*_*i *_+ *D*_*j *_+ (*LD*)_*ij *_+ *ε*_*ijk*_

where *L*_*i *_is the fixed effect of the *i*^th ^hemiclone line (*i *= 1,2,...,40), *D*_*j *_is the fixed effect of the *j*^th ^DIET (yeast-limited or yeast-unlimited), (*LD*)_*ij *_is the hemiclone LINE × DIET (genotype-by-environment) interaction, and *ε*_*ijk *_is the residual variance.

### Analysis of the cost of immunological deployment

Physiological costs of immunological deployment are recognized as a decline in fitness trait values as a consequence of immune system activation. Evolutionary costs of deployment are present if genotypes vary in the physiological cost experienced. We evaluated both of these costs using multivariate, repeated measures analyses. We first compared the natural-log transformed counts of emerging offspring of females receiving an injection of heat-killed bacteria and sterile needle injected and uninjected controls. The multivariate analysis of variance (MANOVA) was carried out by implementing the contrast function in JMP. The contrast response design creates an M-matrix comparing post-injection fecundity (fecundity in vials 4, 5, 6, and 7) to pre-injection fecundity (fecundity in vial 3) for each of the four days post injection. Independent variables in the model included the main effects of hemiclone line, diet and injection in addition to all of their two- and three-way interactions. We also included the effects of block, injector and the number of females in the vial. This last effect was included due to the attrition of flies during the experiment due primarily to escape during transfer but also due to death. In addition to examining results from the full model, tests of each column of the M-matrix compared pre-injection fecundity with fecundity 28, 48, 72 and 96 hours post-injection.

We also had fecundity estimates for the first 28-hours post injection for females receiving an injection of live bacteria. We examined the potential deployment costs associated with the response to live bacteria restricted to a single day post-injection using the same contrast design within the MANOVA platform described above. In this analysis, the response design matrix is a vector of pre-challenge fecundity (vial 3) contrasted with fecundity in the first 28 hours after challenge (vial 4). The independent variables in this analysis are the same as described above for the full analysis across all time periods post-injection. However, in this analysis we constructed a set of *a priori *orthogonal contrasts designed to test specific hypotheses concerning the effects on fecundity of the different injections. The first contrast (*C1*) compared pre-injection and post-injection fecundity between females receiving an injection with live bacteria versus females that received an injection with heat-killed bacteria. This contrast tests whether changes in fecundity following injection differ depending on whether the flies received living or dead bacteria. The second contrast (*C2*) compares the combined means of females receiving bacteria (either living or dead) with females that received a sterile wound. This contrast tests whether costs associated with a response to bacteria can be distinguished from costs associated with wounding. Results from this analysis are only meaningful if the comparison in *C1 *was not significant. The third contrast (*C3*) compares the combined means of females that received an injection of any type (live bacteria, dead bacteria, or a sterile wound) to uninjected females. Provided that the previous contrasts were not significant, *C3 *tests the effect of wounding on changes in fecundity following injection.

## Authors' contributions

KAM and CPY coordinated and carried out the experiment. KAM analyzed the results and drafted the manuscript. All authors participated in outlining the experimental design and read and approved the final manuscript.
